# Adherence to Mediterranean Diet, Malnutrition, Length of Stay and Mortality in Elderly Patients Hospitalized in Internal Medicine Wards

**DOI:** 10.3390/nu11040790

**Published:** 2019-04-05

**Authors:** Aurelio Lo Buglio, Francesco Bellanti, Cristiano Capurso, Annalisa Paglia, Gianluigi Vendemiale

**Affiliations:** Department of Medical and Surgical Sciences, University of Foggia, viale Pinto 1, 71122 Foggia, Italy; aurelio.lobuglio@unifg.it (A.L.B.); cristiano.capurso@unifg.it (C.C.); annalisapaglia@gmail.com (A.P.); gianluigi.vendemiale@unifg.it (G.V.)

**Keywords:** elderly, Mediterranean diet, body composition, circulating interleukins

## Abstract

This investigation aimed to explore the adherence to a Mediterranean Diet and its relationship with length of stay and in-hospital mortality, circulating interleukins, body composition, and frailty, in elderly patients hospitalized in internal medicine wards. Thus, a cross-sectional study in 194 acute hospitalized, community-dwelling elderly patients was performed. Adherence to a Mediterranean Diet was evaluated by the Italian Mediterranean Index (IMI). Length of stay, but not in-hospital mortality rate, was higher in patients with a low IMI score, as compared to subjects with high IMI score. Markers of systemic inflammation, as well as circulating interleukin-6 and tumor necrosis factor alpha, were higher in patients with a low IMI score, with respect to patients with high IMI score. Furthermore, patients with low IMI score had increased fat mass and reduced lean mass, together with a higher prevalence of frailty, as compared to those presenting with high IMI score. In a multivariate logistic regression model, an IMI score < 3 resulted as an independent predictor of longer length of stay. In conclusion, low adherence to a Mediterranean Diet in elderly patients hospitalized in internal medicine wards is associated with higher length of stay and related to unfavorable changes in circulating pro-inflammatory markers and body composition.

## 1. Introduction

The Mediterranean Diet (MD) is characterized by a high intake of “healthy” components, such as vegetables, fruits, cereals, legumes, extra-virgin olive oil, and fish, a moderate consumption of red wine, and a reduced intake of high-fat milk, dairy products, meat, and meat products [1,2]. The MD is of interest for health not only because it is associated with lower mortality from cardiovascular diseases [3,4], but also because it is of benefit to other conditions, such as obesity, type 2 diabetes, neurodegenerative diseases, and certain cancers [5,6,7,8]. The beneficial effects of MD may be exerted through several mechanisms, including anti-inflammatory, antioxidant, hypotensive, hypolipidemic and insulin-sensitizing properties [9,10,11].

Even though the MD is considered the general dietary pattern of the Mediterranean basin, the westernization of eating habits led to a significant decrease in MD adherence from the 1960s to the early 21st century [12]. In fact, despite the positive impact of MD on health status and quality of life, particularly in aged subjects, the adherence to such dietary pattern is decreasing even in the Countries that border the Mediterranean Sea [13].

Malnutrition is extremely prevalent in the geriatric population, involving 15–60% of elderly subjects [14,15]. Particularly, the prevalence of malnutrition is higher among the hospitalized elderly, ranging from 39.2% to 56.2% [16,17]. Indeed, a recent Italian study performed in older people hospitalized in medical wards found a prevalence of malnutrition of 23.2%, with 50.3% of patients at risk of malnutrition [18]. However, although numerous pieces of evidence show the effect of malnutrition on clinical outcomes in this context, screening and assessment of nutritional status are not routinely performed [19,20,21,22]. The Mini-Nutritional Assessment (MNA) is an easy-to-use screening tool to check the nutritional status in the elderly [23]. Elderly subjects diagnosed as malnourished by the MNA show an increased risk of in-hospital mortality and longer length of stay [24]. The association between malnutrition and MD adherence needs to be extensively studied in aged subjects.

Acute conditions which require hospitalization are characterized by increased levels of systemic inflammation. These factors can lead to a decrease in appetite and food consumption, since inflammation is one of the most important causes of malnutrition [25,26]. On the other side, malnutrition can induce an increase in pro-inflammatory indices, favoring a chronic inflammatory state [27]. Furthermore, aging is characterized by a state of low-grade chronic inflammation, associated with high level of acute phase proteins [28,29], as well as pro-inflammatory cytokines, such as interleukin 6 (IL-6) and tumor necrosis factor α (TNF-α) [30,31,32,33]. This condition, defined as “inflammaging”, is associated with increasing mortality and morbidity in older people, and can lead to a decrease in appetite and food consumption (defined as “anorexia of aging”) [25,26].

Aging is also associated with changes in body composition characterized by a progressive increase in fat mass and loss of lean mass, with reduced muscle mass [34]. Additionally, body fat distribution in the elderly presents with unfavorable metabolic features as compared to young people, related to the increase in visceral adiposity [35,36]. These changes play an important role in the inflammatory state, since visceral fat accumulation is associated with an increase in circulating pro-inflammatory cytokines [37]. Furthermore, a low skeletal muscle mass is associated with high levels of C-reactive protein and circulating markers of oxidative stress [30,38,39].

To our knowledge, the association between these biomarkers of inflammation and MD adherence has not been explored in hospitalized elderly. In this scenario, it is important to ascertain the degree of adherence to MD with useful tools, such as validated dietary scores [40].

The aim of this study is to describe the adherence to MD in elderly patients admitted to internal medicine wards, and to investigate its association with nutritional status, length of stay or in-hospital mortality. Furthermore, we also want to assess the impact of MD adherence on circulating markers of inflammation and body composition.

## 2. Materials and method

### 2.1. Study Population and Design

The study was conducted at the Department of Medicina Interna Universitaria, “Ospedali Riuniti” in Foggia (Italy). We recruited 194 consecutive patients aged 65 years or older, admitted to our ward from May 2017 to April 2018. The exclusion criteria were the following: Dysphagia, active cancer, severe cognitive impairment (assessed with a Mini-Mental State Examination score ≤ 9 points), inability to comply with the study protocol or to provide written informed consent. The study was approved by our Institutional Review Board at the Ospedali Riuniti in Foggia and performed according to the Declaration of Helsinki. All patients gave written informed consent.

Adherence to MD was assessed using the Italian Mediterranean Index (IMI), whose score is calculated from the intake of 11 items: High intakes of six typical Mediterranean foods (pasta; typical Mediterranean vegetables; fruits; legumes; olive oil; and fish); low intakes of four non-Mediterranean foods (soft drinks, butter, red meat, and potatoes); and moderate alcohol intake. One point was assigned if the intake of typical Mediterranean foods were in third tertile; all other intakes received 0 points. One point was assigned if the consumption of non-Mediterranean foods was in the 1st tertile. Ethanol consumption received 1 point for intake up to 12 g·d^−1^; abstainers and persons who consumed >12 g·d^−1^ received 0 points [41]. Patients were divided into three groups according to tertiles of IMI score.

Nutritional status was evaluated through the MNA, a validated tool composed of 18 questions regarding anthropometric, general, dietetic, and subjective evaluation. MNA identifies patients with malnutrition (score < 17), at risk of malnutrition (score 17–23.5), and well-fed (score > 23.5) [42]. In this study, poor nutritional status was defined as being either malnourished or at risk of malnutrition. Cognitive function was assessed through the Mini-Mental State Examination (MMSE), an 11-item questionnaire which assesses orientation, memory, attention, ability to follow verbal instructions and produce written language, and visuospatial skills; MMSE is provided of documented validity and reliability [43]. Function autonomy was measured using the activities of daily living (ADL) scale and the instrumental activities of daily living (IADL) scale. ADL considers basic daily activities, such as bathing, getting dressed or eating; IADL scale assesses complex activities, such as managing with public transportation, finances, or shopping. ADL score ranges from 0 to 6 points, while the IADL score ranges from 0 to 8 points [44,45]. Depressive symptoms were evaluated using the Geriatric Depression Scale short-form (GDS-SF), a practical 15-item tool validated in elderly people, which classifies subjects into the following categories: No depression (0 to 5 points), mild depression (6 to 9 points), and severe depression (10 to 15 points) [46]. Frailty status was evaluated according to Fried’s criteria (Weight loss, Exhaustion, Low physical activity, Walk time, and Grip strength): Patients without any of five criteria were defined as No-frail (NFR), those with 1 to 2 criteria as Pre-frail (PFR), and those three or more criteria as Frail (FR) [47].

Data related to in-hospital death and length of stay (days) were recorded.

### 2.2. Biochemical Analysis, Anthropometric Measurements and Body Composition Evaluation

A blood sample was taken at the time of admission for the determination of hemoglobin (Hb), white blood cell (WBC), lymphocytes, serum glucose, albumin, total cholesterol, creatinine and triglycerides, erythrocyte sedimentation rate (ESR), ferritin, C-reactive protein (CRP), uric acid and creatinine. Neutrophil/Lymphocytes ratio (NLR) was calculated by dividing the neutrophil’s number by lymphocyte’s number. The concentrations of serum cytokines and growth factors, including IL-1α, IL-1β, IL-2, IL-4, IL-6, IL-8, IL-10, Tumor Necrosis Factor-α (TNF-α), Interferon-γ (IFN-γ), Monocyte Chemoattractant Protein-1 (MCP-1), Epidermal Growth Factor (EGF), and Vascular Endothelial Growth Factor (VEGF), were measured using the EV 3513 cytokine biochip array and competitive chemiluminescence immunoassays (Randox Laboratories Ltd., Crumlin, UK), according to the manufacturer’s instructions, using the Randox Evidence Investigator (Molloy RM et al., Clin Chem Lab Med, 2005).

Height, body weight, and waist, arm, calf and leg circumference were measured according to standardized procedures. Body weight in bed-ridden patients was measured by using a LikoScale350 system (LikoAB, Luleå, Sweden). Body mass index (BMI) was calculated as the ratio between weight in kilograms and the square of height in meters. Body composition was assessed by bioelectrical impedance using a BIA 101-F device (Akern/RJL, Florence, Italy), as previously reported [48].

### 2.3. Statistical Analysis

Data were expressed as count and percentages/interquartile range (IR) for qualitative values, and as mean ± standard deviation of the mean (SDM) for quantitative variables. Gaussian distribution of the samples was evaluated by the Kolmogorov-Smirnov test. Comparison among groups for continuous variables was performed using one-way ANOVA or Kruskall-Wallis tests for parametric or non-parametric distribution respectively. The Tukey test was used for the post hoc analysis. Nominal and categorical variables were analyzed by the Pearson’s Chi-Squared test.

The odds ratio (OR) and the 95% confidence interval (CI) were calculated. Univariate binary logistic regression analysis was used to analyze the association between nutritional status (malnourished with MNA < 17 points, well-fed with MNA ≥ 17 points) and MD adherence (low if IMI < 3 points, high if IMI > 6 points), cognitive status (impairment with MMSE < 24 points, normal with MMSE ≥ 24 points), independence (independent if ADL 6 points or IADL 8 points, dependent if ADL < 6 points or IADL < 8 points), depressive symptoms (depression if GDS-SF > 5 points, no depression if GDS-SF ≤ 5 points), and frailty (non-frail < 3 criteria, frail ≥ 3 criteria). Further multivariate binary logistic regression analysis was performed to identify (1) the association of mortality with low MD adherence, malnutrition, cognitive status, physical performance, depressive symptoms, and frailty as covariates, and (2) the association of malnutrition with low MD adherence, cognitive status, physical performance, depressive symptoms, and frailty as covariates. The selection of covariates in the multivariate analysis was performed by back-ward selection, using *p* < 0.1 as a cut-off, with age and genre forced into the model. All tests were 2-sided, and *p* values < 0.05 were considered statistically significant. Statistical analysis was performed with the Statistical Package for Social Sciences version 20.0 (SPSS, Inc., Chicago, IL, USA) and the package Graph-Pad Prism 6.0 for Windows (GraphPad Software, Inc., San Diego, CA, USA).

## 3. Results

194 patients (77.7 ± 8 years old) were enrolled: Of these, 45 (23.2%) were included in the Tertile I (score 0–3), 84 (43.3%) in the Tertile II (score 4–5), and 55 (33.5%) in the Tertile III (Score 6–11). Baseline clinical and biochemical characteristics of patients according to the study groups are reported in Table 1.

Patients included in the Tertile II and III groups were older than WF ones; furthermore, high MD adherence was observed more frequently in female rather than male patients. No differences were found in the rate of patients with comorbidities between all the groups studied. When compared to Tertile III, Tertile II and I patients presented with lower hemoglobin, lymphocytes, serum albumin and total cholesterol levels; post hoc analysis showed no differences between Tertile II and Tertile I groups. Serum creatinine level, glycaemia, and triglycerides were similar among all the groups studied.

Nutritional and cognitive status, physical performance, and depressive symptoms were significantly different among the groups studied (Table 2). Post hoc analysis resulted in lower MNA, MMSE, ADL and IADL scores in Tertile I group compared to Tertile II and Tertile III; GDS-SF score was higher in Tertile I group than Tertile III, but there were no significant differences between Tertile I and II (Table 2).

Frailty status was diagnosed in 70.6% of patients (FR, age 80.1 ± 7.7), while 17% were Pre-frail (PFR, age 73.3 ± 7.7), and 12.4% were Not-frail (NFR, age 72.8 ± 4.8). All the Tertile I patients were frail, while the prevalence of frailty in Tertile II and III groups was 89.3% and 29.2%, respectively. Prevalence of each frailty parameter (according to Fried definition) was higher in Tertile I and II groups rather than Tertile III patients (Table 2).

### 3.1. MD Adherence, Length of Stay and in-Hospital Mortality

**Figure 1:**
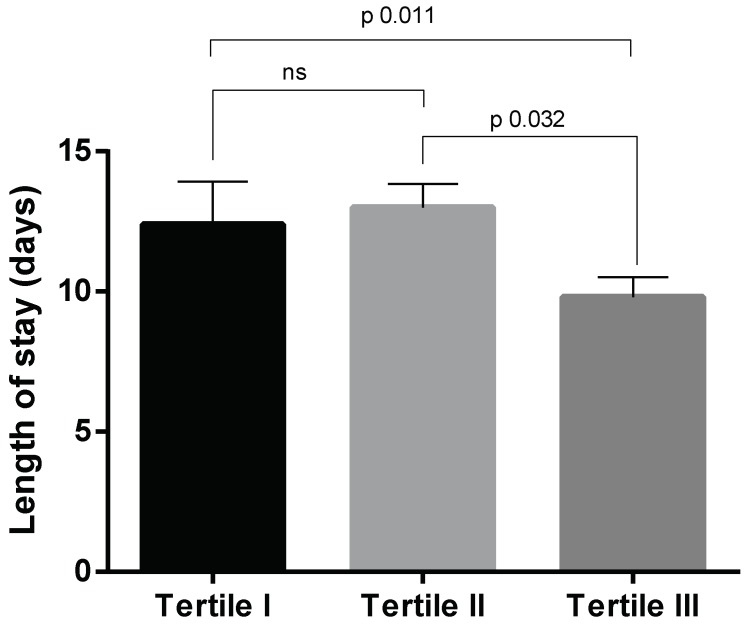
Length of stay in hospitalized elderly patients enrolled in this study and stratified according to the mini nutritional assessment at admission in well-fed (WF, *N* = 65), at risk of malnutrition (RM, *N* = 84), and malnourished (M, *N* = 45). Data are expressed as mean ± SEM. Statistical differences were assessed by one-way ANOVA and Tukey as post hoc test. *p* Values < 0.05 were considered statistically significant.

As shown in Figure 1, length of stay in hospitalized elderly subjects who were included in the Tertile I and II was longer than Tertile III patients; post hoc analysis did not evidence any difference between Tertile I and II groups. In-hospital mortality rate was 5.1% (*N* = 10). Two deaths (3.1%) were registered in the Tertile III group, while 3 (3.6%) were reported in Tertile II and 5 (11.1%) in the Tertile I group (χ2 4.27, *p* 0.118).

### 3.2. MD Adherence and Circulating Markers of Inflammation

Markers of systemic inflammation, such as NLR, ESR, CRP and ferritin, were higher in Tertile I patients than other groups, as shown in Table 3. Particularly, post hoc analysis showed that NLR, ESR and ferritin were higher in Tertile I group rather than Tertile II and III, while no difference between Tertile I and Tertile II was reported. CRP in Tertile I and II was higher than Tertile III group; furthermore, Tertile I patients showed higher values than Tertile II ones.

Table 3 also summarizes the results related to serum levels of 12 different cytokines and growth factors evaluated in hospitalized elderly patients. Higher levels of IL-6 and TNF-α were reported in Tertile I compared to Tertile II and III subjects; post hoc analysis showed that circulating IL-6 (but not TNF-α) was more elevated in Tertile II rather than Tertile III patients. No further differences were found for the other cytokines and growth factors analyzed.

### 3.3. Nutritional Status, Anthropometric Parameters and Body Composition

Table 4 summarizes results related to anthropometric measures and bioimpedentiometry, taking into account the adherence to MD at admission of patients enrolled. BMI, as well as arm, thigh, waist, and calf circumference, were different in all the considered groups. Post hoc analysis showed that all these measures were lower in Tertile I as compared to Tertile II and III patients, while there were no significant differences between Tertile II and Tertile III groups.

Body composition was different among groups concerning the percentage of FM, FFM and MM. Post hoc analysis showed that the percentage of FFM and MM was lower in Tertile I compared to Tertile II and III patients, and that of Tertile II was lower than Tertile III group. On the contrary, FM percentage was higher in Tertile I than Tertile II and III groups, and that of Tertile II was higher than Tertile III. There were no differences among groups in the percentage of TBW, ECW and ICW (Table 4).

### 3.4. Factors Associated with in-Hospital Mortality in Elderly Patients

**Figure 2:**
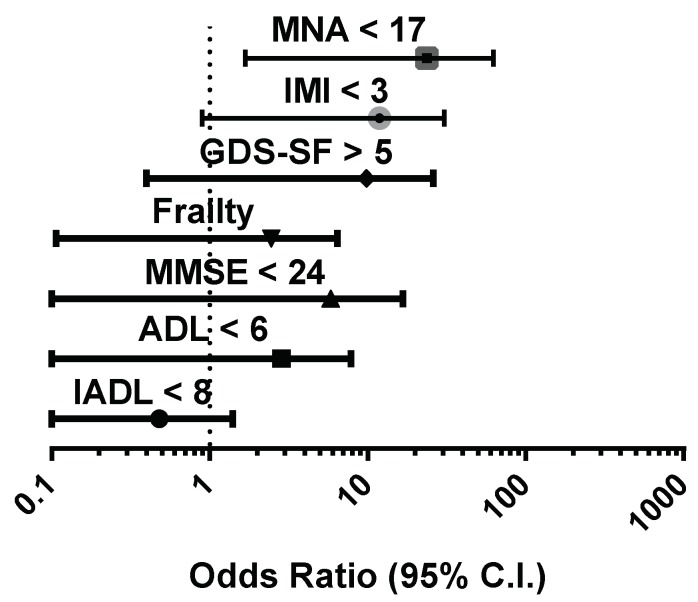
Odds ratios of factors associated with in-hospital mortality in a multivariate logistic regression model applied in the entire cohort studied. MNA, mini nutritional assessment; IMI, Italian Mediterranean index; GDS-SF, geriatric depression scale-short form; ADL, activities of daily living; IADL, instrumental activities of daily living; MMSE, mini-mental state examination.

A multivariate analysis was performed to verify the most important factors associated with the occurrence of in-hospital mortality (Figure 2), showing that MNA score < 17 (malnutrition/risk of malnutrition) was the only independent predictor (OR: 6.556, *p* = 0.006).

**Figure 3:**
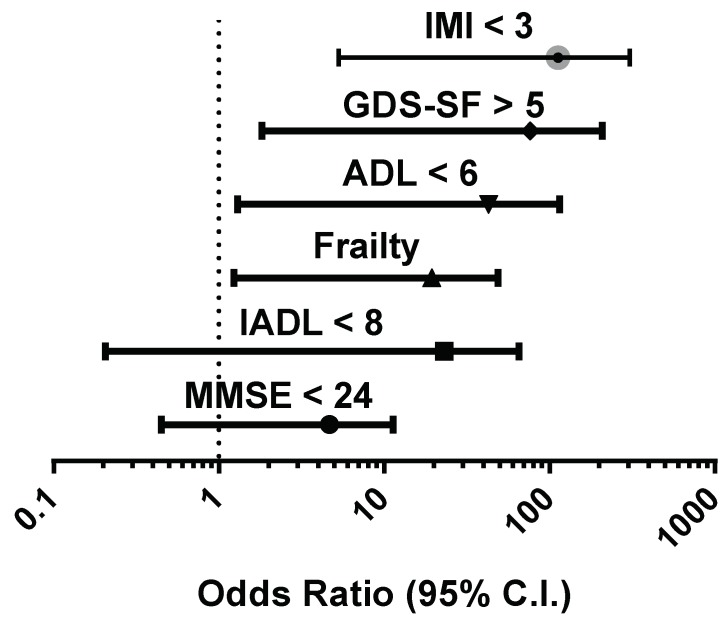
Odds ratios of factors associated with malnutrition (MNA score < 17) in a multivariate logistic regression model applied in the entire cohort studied. IMI, Italian Mediterranean index; GDS-SF, geriatric depression scale-short form; ADL, activities of daily living; IADL, instrumental activities of daily living; MMSE, mini-mental state examination.

A further multivariate analysis was performed to investigate the most important factors associated with the presence of malnutrition (Figure 3) and showed that the scarce adherence to MD was the best independent predictor (OR: 28.435, *p* = 0.008), followed by the presence of depressive symptoms (OR: 19.523, *p* = 0.019), loss of physical performance (OR: 12.226, *p* = 0.028), and frailty status (OR: 7.792, *p* = 0.029).

## 4. Discussion

To our knowledge, this is the first study reporting that hospitalized elderly patients who present with scarce adherence to MD at admission, as determined by the IMI, are subjected to a longer length of stay. Furthermore, hospitalized elderly patients with low MD adherence exhibit circulating pro-inflammatory markers and alterations in body composition. Finally, low adherence to MD is the main determinant of malnutrition in these patients.

The MD is a long-established dietary pattern with a positive impact on health, quality of life, and longevity [2,49]. Several studies investigated the adherence to the MD in populations of different ages, applying different validated scores [50,51,52,53]. Only a few studies focused on MD adherence in the elderly [54,55]. According to these previous reports, our data confirm that the higher level of adherence to the MD is observed in older subjects. Nevertheless, the report that the majority of our sample was included in Tertiles I and II supports the recent evidence indicating a progressive decline of this dietary pattern, even in Mediterranean areas.

The present study shows that low adherence to MD is related to poor nutritional status. Indeed, elderly people at risk of malnutrition present with minimal daily consumption of fruits and vegetables [56]. On the contrary, adherence to MD is related to adequate nutritional status in aged people [57].

Aging is related to unfavorable variations in body composition, such as loss of muscle mass and increased fat mass. A scarce adherence to MD can contribute to these modifications, especially the loss of skeletal muscle mass [58,59]. The present data show that elderly patients with low adherence to MD present with lower muscle mass and higher fat mass. We could not exclude that these results are also related to age and genre, since patients in the Tertile I or II of IMI score were mostly women with advanced age with respect to Tertile III ones. Protein intake in the traditional MD is 20% lower than a Western diet; particularly, most of the protein comes from legumes and cereals, while animal proteins are 50–60% lower. Other than quantity, protein quality may mediate the beneficial effects of MD on body composition, since the restriction of branched-chain amino acids may be related to reduced fat mass [60]. However, the exact mechanism by which adherence to the traditional MD exerts its effects on body composition is worth of future investigation.

Low adherence to MD is associated with high levels of several circulating inflammatory markers in both adults and adolescents [61]. According to this, we found higher levels of markers of systemic inflammation in elderly patients included in the Tertiles I and II of IMI score. In addition, these patients presented with higher circulating levels of TNF-α and IL-6. Whether MD adherence could affect the secretion of acute phase proteins or cytokines/growth hormones is still a matter of debate. Nevertheless, it has been previously demonstrated that adherence to MD is inversely related to circulating IL-6 [62]. The MD is well-recognized to exert anti-inflammatory effects, as concluded by several systematic reviews and meta-analyses [63,64]. This may be related to the synergic properties of vegetables, fruits, and olive oil, as well as specific nutrients, such as *α*-tocopherol, ascorbic acid, *β*-carotene, flavonoids, and *ω*-3 polyunsaturated fatty acids.

It is worth to note that high levels of IL-6 and TNF-α increase the catabolic activity in skeletal muscle, promoting proteolysis and loss of muscle mass [65,66,67]. On the other side, in the elderly these cytokines lead to a lower food intake through various mechanisms, such as delayed gastric emptying and reduction of intestinal motility (“anorexia of aging”) [68,69]. We could suggest that the increased cytokines would represent a possible mechanism which mediates muscle mass depletion in aged people with low adherence to MD.

If our study reports that elderly patients hospitalized in internal medicine wards presenting with high MD adherence at admission had a reduced length of stay, we could not find any positive impact of MD adherence on in-hospital mortality rate. Interestingly, a poor nutritional status was a strong predictor of mortality in our study population. This is consistent with previous data showing a higher mortality among malnourished hospitalized patients in internal medicine wards, as well as among critically ill patients [70,71]. Several studies report a consistent prevalence of malnutrition at admission, particularly in elderly patients [24,72]. In an Italian retrospective analysis, 24.6% of elderly hospitalized patients were malnourished, and 28.2% were at risk of malnutrition [73]. A further study on 623 hospitalized patients reported that 24% were malnourished, compared to 58% at risk of malnutrition [74]. Malnutrition is most commonly observed in aged subjects. Age-related malnutrition depends on several mechanisms, such as lack of physical activity, poor appetite, loneliness and sense of neglect [34]. Although previous studies have shown the impact of nutritional status on length of stay and in-hospital mortality [75,76], there is little information about elderly patients hospitalized in internal medicine wards. The nutritional status of aged subjects is related to morbid conditions as cancer, dementia, and dysphagia [77]. However, these comorbidities were considered as exclusion criteria in the present study. A further important finding of this study is related to the best predictor of malnutrition at admission in hospitalized elderly patients, which has been identified as the adherence to MD. It is conceivable that reduced healthy food intake could be related to unsatisfying response to medical therapies. The association between MD adherence, malnutrition and poor clinical outcomes confirms the importance of early nutritional assessment at admission in internal medicine wards.

The strengths of this study consist of the prospective design and in the assessment of MD adherence at hospital admission by the IMI, which is a detailed dietary questionnaire [41]. Nevertheless, this study presents the main limitations. First, it is a single-center study with a small sample size, which restricted the subgroup analysis. Moreover, this study did not investigate the association between MD adherence and the underlying disease that led to hospitalization. A further limitation of the study is that consumption evaluation was based on a single dietary assessment in which patients were asked about eating behaviors over the preceding year, even though dietary patterns are more consistent than single nutrients as indicators of long-term typical diet [78]. Moreover, in our study we did not estimate confounding factors, such as socio-economic and family status, which could partially impact the results.

In conclusion, the present study points out that two-thirds of elderly patients hospitalized in internal medicine wards present with low adherence to MD at admission, and extensive length of stay with respect to those with high adherence. These subjects show unfavorable changes in the pro-inflammatory status and body composition, as well as poor nutritional state, which could contribute to worse clinical outcomes. These results point out the importance of MD and nutritional status evaluation, in order to promptly implement interventions with adequate nutritional supplementation.

## Figures and Tables

**Table 1:** Clinical and biochemical characteristics of patients at baseline, according to study groups.

	Tertile I (score 0–3)	Tertile II (score 4–5)	Tertile III (score 6–11)	*p* Value
	**n. 45 (23.2%)**	**n. 84 (43.3 %)**	**n. 65 (33.5%)**	
**Age (years)**	73.8 ± 7.5	78.8 ± 7.1	81.3 ± 8.3	**<0.001**
**Genre M/F (n, %)**	39/26 (60/40)	32/52 (38/62)	12/31 (31/69)	**0.004**
**Co-morbidities ≥ 3 (n, %)**	16 (24.6)	25 (29.8)	17 (37.8)	0.333
**Haemoglobin (g/dL)**	11.4 ± 1.5	10.8 ± 1.9	12.6 ± 2.1	**<0.001**
**WBC (n/mm^3^)**	5968.5 ± 1230.2	8508.9 ± 900.9	7480.9 ± 1104.0	0.061
**Lymphocytes (n/mm^3^)**	1129.5 ± 616	1459.2 ± 802	1543.7 ± 620	**0.013**
**Glucose (mg/dL)**	126.7 ± 77.3	114.4 ± 48.9	112.5 ± 35.7	0.349
**Albumin (g/dL)**	2.8 ± 0.5	3.0 ± 0.5	3.1 ± 0.6	**<0.001**
**Total Cholesterol (mg/dL)**	142.9 ± 42	140.2 ± 45.6	163.6 ± 45.1	**0.005**
**Creatinine (mg/dL)**	1.1 ± 0.4	1.3 ± 0.8	1.3 ± 0.8	0.159
**Triglycerides (mg/dL)**	116.7 ± 56.5	114.4 ± 52.7	104.6 ± 39.3	0.463

Statistical differences were assessed by one-way analysis of variance or Pearson’s Chi-squared test. *p* values < 0.05 were considered statistically significant (in bold). WBC, white blood cells.

**Table 2:** Nutritional status, cognitive status, physical performance, depression and frailty status in patients at baseline, according to study groups.

	Tertile I (score 0–3)	Tertile II (score 4–5)	Tertile III (score 6–11)	*p* Value
	n. 45 (23.2%)	n. 84 (43.3%)	n. 65 (33.5%)	
**MNA (score)**	12.3 [7.3–19.4]	18.7 [10.2–22.1]	22.4 [13.7–28.9]	**<0.001**
**MMSE (score)**	14.1 [9.7–21.3]	21.4 [15.5–25.3]	26.3 [23.9–28.0]	**<0.001**
**ADL (score)**	1.9 ± 2.0	4.3 ± 2.0	5.7 ± 0.9	**<0.001**
**IADL (score)**	1.2 ± 0.6	3.9 ± 2.5	6.2 ± 2.1	**<0.001**
**GDS-SF (score)**	5.0 [3.2–7.7]	5.0 [2.0–8.0]	2.0 [0.0–4.0]	**<0.001**
**Frail (N, %)**	45 (100%)	75 (8.3%)	19 (29.2%)	**<0.001**
**Fried’s score items**				
**Weight loss (N, %)**	27 (60.0%)	30 (35.7%)	2 (3.1%)	**<0.001**
**Exhaustion (N, %)**	42 (93.3%)	74 (88.1%)	33 (50.8%)	**<0.001**
**Low physical activity (N, %)**	43 (95.5%)	63 (75%)	18 (27.7%)	**<0.001**
**Low walking speed (N, %)**	45 (100%)	68 (80.9%)	18 (27.7%)	**<0.001**
**Low grip strength (N, %)**	45 (100%)	73 (86.9%)	22 (33.8)	**<0.001**

Statistical differences were assessed by one-way analysis of variance or Pearson’s Chi-squared test. *p* values < 0.05 were considered statistically significant (in bold). MNA, mini-nutritional assessment; MMSE, mini-mental state examination; ADL, activity of daily living; IADL, instrumental activity of daily living, GDS-SF, geriatric depression scale short form.

**Table 3:** Markers of systemic inflammation, circulating cytokines and growth factors in patients at baseline, according to study groups.

	Tertile I (score 0–3)	Tertile II (score 4–5)	Tertile III (score 6–11)	*p* Value
	n. 45 (23.2%)	n. 84 (43.3%)	n. 65 (33.5%)	
**NLR**	6.8 ± 4.8	4.7 ± 3.2	3.1 ± 1.6	**<0.001**
**ESR (mm/h)**	53.5 [28.5–76.7]	43.5 [25–65.5]	29.0 [12.0–57.0]	**0.011**
**CRP (ng/mL)**	26.8 [13.0–35.7]	15.3 [6.5–23.2]	4.4 [1.8–4.4]	**<0.001**
**Ferritin (ng/mL)**	168 [64.5–394.0]	130 [46.0–398.0]	86 [44.5–154.2]	**0.017**
**IL-1β (pg/mL)**	0.83 ± 0.25	0.92 ± 0.31	0.72 ± 0.11	0.233
**IL-2 (U/mL)**	1.21 ± 0.99	0.93 ± 0.46	0.69 ± 0.19	0.112
**IL-6 (pg/mL)**	21.66 ± 5.16	11.94 ± 5.77	3.37 ± 1.72	**<0.001**
**IL-8 (pg/mL)**	118.52 ± 46.09	89.92 ± 21.14	97.14 ± 28.72	0.585
**IL-10 (pg/mL)**	0.66 ± 0.65	2.61 ± 1.5	2.23 ± 1.14	0.103
**TNF-α (pg/mL)**	6.43 ± 3.2	4.50 ± 1.92	13.18 ± 1.97	**0.001**
**IL-4 (pg/mL)**	0.84 ± 0.22	0.82 ± 0.17	0.52 ± 0.32	0.344
**IL-1α (pg/mL)**	0.52 ± 0.08	0.54 ± 0.05	0.53 ± 0.06	0.129
**VEGF (pg/mL)**	264.38 ± 48.87	262.40 ± 32.66	289.81 ± 35.28	0.858
**IFN-γ (pg/mL)**	2.38 ± 1.24	2.16 ± 1.06	1.94 ± 0.45	0.565
**EGF (pg/mL)**	18.92 ± 3.84	13.17 ± 2.10	14.12 ± 1.48	0.256

Statistical differences were assessed by one-way analysis of variance. *p* values < 0.05 were considered statistically significant (in bold). NLR, neutrophils/lymphocytes ratio; ESR, erythrocytes sedimentation rate; CRP, C-reactive protein; IL, interleukin; TNF-α, Tumor Necrosis Factor- α, VEGF, Vascular Endothelial Growth Factor; IFN-γ, Interferon-γ, EGF, Epidermal Growth Factor.

**Table 4:** Anthropometric measures and bioimpedentiometric parameters in patients at baseline, according to study groups.

	Tertile I (score 0–3)	Tertile II (score 4–5)	Tertile III (score 6–11)	*p* Value
	n. 45 (23.2%)	n. 84 (43.3%)	n. 65 (33.5%)	
**BMI (Kg/m^2^)**	24.7 ± 4.9	27.9 ± 5.5	28.3 ± 4.6	**0.001**
**Arm circumference (cm)**	23.2 ± 4.4	26.7 ± 5.3	28.6 ± 4.4	**<0.001**
**Thigh circumference (cm)**	36.9 ± 7.1	43.6 ± 7.3	46.2 ± 11.1	**<0.001**
**Waist circumference (cm)**	92.4 ± 17.1	144.9 ± 16.8	105.4 ± 15.1	**<0.001**
**Calf circumference (cm)**	28.4 ± 6.8	32.2 ± 7.4	33.6 ± 5.0	**<0.002**
**FM (%)**	41.7 ± 5.7	37.3 ± 4.8	30.6 ± 2.6	**<0.001**
**FFM (%)**	57.8 ± 6.1	62.6 ± 4.9	69.4 ± 2.6	**<0.001**
**MM (%)**	24.6 ± 2.2	26.9 ± 3.1	33.2 ± 4.5	**<0.001**
**TBW (%)**	53.5 ± 5.8	55.9 ± 12.5	57.2 ± 9.8	0.709
**ECW (%)**	53.5 ± 9.9	63.0 ± 12.3	62.9 ± 9.1	0.057
**ICW (%)**	46.5 ± 9.9	36.9 ± 12.3	37.0 ± 9.1	0.059

Statistical differences were assessed by one-way analysis of variance. *p* values < 0.05 were considered statistically significant (in bold). BMI, body mass index; FM, fat mass; FFM, free-fat mass; MM, muscle mass; TBW, total body water, ECW, extracellular water; ICW, intracellular water.
